# Prevalence of Anaemia and Associated Factors among Children below Five Years of Age in Cape Verde, West Africa

**Published:** 2014-12

**Authors:** Rosa M.L. Semedo, Marta M.A.S. Santos, Mirian R. Baião, Ronir R. Luiz, Gloria V. da Veiga

**Affiliations:** ^1^Department of Nutrition, Federal University of Rio de Janeiro; ^2^Department of Public Health Studies, Federal University of Rio de Janeiro, Brazil

**Keywords:** Anaemia, Child, Risk factors, Socioenvironmental conditions, West Africa

## Abstract

This study estimated the prevalence of anaemia and associated factors in a probability sample of 993 children aged 6-59 months in Cape Verde, West Africa. Odds ratio (OR) and 95% confidence interval (95% CI) were estimated from a hierarchical model for multiple analysis to assess the association between anaemia and explanatory variables. The prevalence of anaemia was 51.8% (95% CI 47.7-55.8). Children who resided within poor household conditions (OR 1.99; 95% CI 1.06-3.71) were below 24 months of age (OR 3.23; 95% CI 2.03-5.15) and recently experienced diarrhoea (OR 1.58; 95% CI 0.99-2.50) were at high risk of anaemia. Anaemia should be considered a serious public-health concern in Cape Verde, mainly for children below 24 months. Further, special consideration should be given to children who have experienced recent diarrhoea and belong to families residing in poor household conditions.

## INTRODUCTION

Iron-deficiency anaemia is the most prevalent nutritional disorder in the world, affecting both developed and developing countries. The World Health Organization has estimated that approximately 1.6 billion people (i.e. close to one-fourth of the world's population) have anaemia; Africa is the most affected region. Anaemia is most prevalent among pregnant women and children below five years of age and is particularly prevalent during the first two years of life. Around 60% of African children below five years of age have anaemia (1). In sub-Saharan Africa, the prevalence of anaemia among preschool children ranges from 42% in Swaziland to 91% in Burkina Faso (2).

The Second Demographic and Reproductive Health Survey (3), conducted in 2005, showed that 52% of children aged ≤5 years in Cape Verde, West Africa, were anaemic. In a 1996 survey, prevalence of anaemia in this age-group was 70% (4). Despite the apparent decrease, the prevalence of anaemia is still alarmingly high and is a serious public-health concern (5) because of its capacity to impair cognitive and psychomotor development (6) and the general health of children. Moreover, no specific preventive or control policies for anaemia in Cape Verde target this age-group, further increasing their vulnerability to this condition.

In developing countries, insufficient dietary iron is considered the primary cause of anaemia in children. However, other factors, such as early weaning, poor health of pregnant women, insufficient safe drinking-water, inadequate hygiene and sanitary conditions, and poverty which increases the likelihood of all the abovementioned factors, may contribute to the development of the disease (7). Additional risk factors of anaemia include glucose-6-phosphate dehydrogenase deficiency, haemoglobinopathies, and infectious diseases, such as malaria, which is endemic in African countries (8).

To date, no studies have explored the possible determinants of anaemia in children in Cape Verde. Considering the high prevalence of childhood anaemia in Cape Verde, its deleterious effects on children's health, possible developmental repercussions, and the absence of specific public health policies addressing this problem, anaemia is a high-priority topic for investigation. Thus, the present study aimed to assess the prevalence of anaemia among Cape Verdean children between 6 and 59 months of age and to identify high-risk subgroups based on the analysis of possible associated factors.

## MATERIALS AND METHODS

### Sociodemographic characteristics of Cape Verde

Cape Verde is a small, archipelagic country in sub-Saharan Africa, with 491,575 inhabitants and a population growth rate of 1.23% per year. Nearly 10% of the population is below five years of age (9). Poverty in Cape Verde is more prevalent in single-parent families headed by women, with large numbers of children (3.7 children per household) living with low-quality sanitation and minimal education. Poverty affects 29% of families with children aged 0-5 year(s) (10). Mortality in under-five children has decreased from 31.9 per thousand in 2000 to 23.7 per thousand in 2009 (11).

### Study design

The data analyzed in this study were derived from the survey on the Prevalence of Anemia and Associated Factors among Children under the Age of 10 Years in Cape Verde (IPAC, 2009), developed by the Ministry of Environment, Rural Development and Marine Resources of Cape Verde.

This was a cross-sectional household-based study with a probabilistic sample of 2,383 children between 6 months and 9 years of age. A two-stage cluster-sampling design was used, based on the “Wellbeing Indicator Questionnaire” (QUIBB) survey (10). The first stage related to the delineation of 11 study domains comprising the 9 inhabited islands of Cape Verde, one of which (the island of Santiago) was divided into 3 domains (Santiago Norte, Praia Urban, and the remainder of Santiago). The second stage corresponded to the locations of the homes, which were classified as urban or rural.

A total of 284 children were excluded from the analysis due to the absence of data on birth date, weight, height, and haemoglobin concentration. Thus, the final sample was composed of 2,099 children, representing 88% of eligible children. The sample analyzed in the present study consisted of a subsample of 993 children aged 6-59 months, who were part of the national survey. This sample-size allows us to estimate the prevalence of anaemia to the age range of about 50%, with an error of about 4.5%, and shows the effect of drawing in 2.0 (12).

### Evaluation of anaemia

Data were collected between May and August 2009. Anaemia was determined by assaying blood haemoglobin (Hb) levels in the collected samples by using a portable haemoglobinometer (HemoCue). The HemoCue is a practical portable device that has been validated and recommended for use in population-based studies. This device is non-invasive requiring only a small volume of blood (10 µL) and confers high sensitivity, specificity, and positive predictive value (13) for evaluation of blood haemoglobin. Blood samples were obtained via finger puncture with disposable lancets by nursing technicians trained by an experienced biotechnician from Central Hospital of Cape Verde according to the protocol described by the HemoCue manufacturer.

Children with haemoglobin levels less than 11 g/dL were considered to be anaemic in accordance with the WHO classification system (5).

### Anthropometry

Anthropometric measurements were conducted by interviewers trained and standardized within the margins of error accepted by the Habitch's criterion (14). Interviewers who failed to achieve the minimal values for reliability and validity underwent further standardization sessions to reach acceptable level. The training was provided by Brazilian researchers from the Federal University of Rio de Janeiro, who constituted the team of advisors for the project. Weight, length (for children aged less than two years), and height (for children over two years of age) were measured. Weight was measured using a Seca electronic platform scale with a capacity of 150 kg and a variation of 50 g. A portable wooden anthropometer for children adapted for this study was used for measuring height while the child was in a standing position. Length was measured using the same anthropometer, with the child in a horizontal position. Height or length was measured twice by the same evaluator, allowing a maximum variation of 0.5 cm between the two measurements; the mean was recorded and used in the analysis.

Nutritional status was classified based on the following indices: weight-for-age z-score (WAZ), height- or length-for-age z-score (HAZ), and weight-for-height or length z-score (WHZ). In accordance with the criteria proposed by WHO (15), nutritional deficits were defined as <−2 z-scores from the WAZ, WHZ, and HAZ ratios, and obesity was defined as ≥2 z-scores from the mean WHZ.

### Socioeconomic, demographic, and child morbidity characteristics

A questionnaire was administered to the child's parents or caregivers during an interview to obtain information on the following variables: (a) child's sex, age, breastfeeding, vaccination, and illness history; (b) socioeconomic and environmental conditions, including parents’ educational level and household conditions.

### Ethical permission

This study was conducted in accordance with the standards set forth in the Declaration of Helsinki, and all procedures involving human subjects were approved by the National Committee of Ethics in Research of Cape Verde. The children's parents or caregivers signed an informed consent statement. Children who presented with anaemia were referred to the nearest health service clinic.

### Statistical analysis

Statistical analyses were carried out taking into account the effect of the sampling design per cluster in the two selection stages (domain and residential location, urban or rural) and sampling expansion corrected by the relative weight, using the complex sample procedure in Statistical Package for Social Sciences (version 19.0) for Windows (SPSS Inc., Chicago, IL, USA). Associations between anaemia and the exposure variables were assessed by logistic regression with an estimation of odds ratios (OR) and a 95% confidence interval (95% CI). We chose the odds ratio to measure associations based on the arguments of Pearce (16). Following this, a hierarchical multivariate analysis was carried out in accordance with a conceptual model that was defined a *priori* and adapted from De Silva *et al*. (17) as shown in the Figure. The hierarchical multivariate analysis included all variables investigated, regardless of their statistical significance in the bivariate analysis because we were interested in the relationships among these anaemia-associated variables. The first dimension of the model included the socioeconomic variables and those relating to environmental conditions, educational level of the head of household, and household conditions. The latter variable was constructed based on the following information for all the households: ownership of house for accommodation, type of accommodation, predominant material used for the roofing and flooring, presence of a kitchen and bathroom, electrical service, waste disposal, residual water disposal, origin of drinking-water, water treatment, energy used in preparing food, and the presence of amenities and appliances (refrigerator, stove, television, automobile, radio, and telephone). These items have been used in other surveys conducted by the National Institute of Statistics in Cape Verde to measure household socioeconomic conditions. These were additionally used in the Welfare Indicators of Cape Verde Families Unified Questionnaire (10), an adaptation of the questionnaire used by the World Bank to monitor poverty reduction strategies, projects, and outcomes (18).

**Figure.**
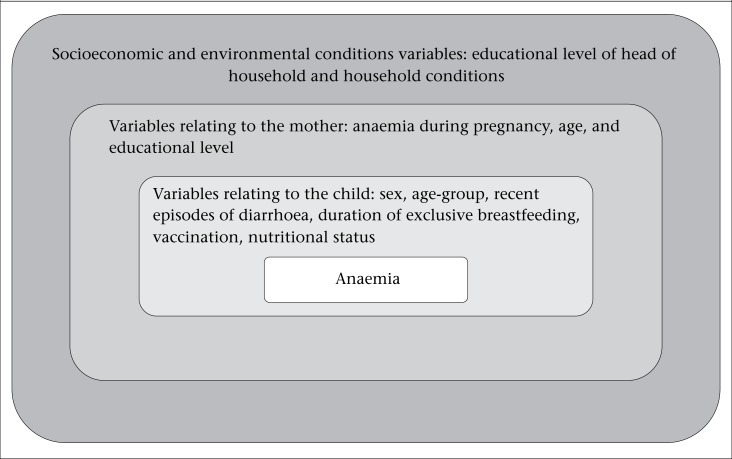
Conceptual hierarchical model adapted from De Silv a *et al.* 2001

Scores ranging from 0 (worst condition) to 5 (best condition) were assigned for each household variable. Thus, based on the sum of all variables, total scores could range from 0 to 39 points; maximum scores reflect ideal conditions for a family in this country. The variable relating to socioenvironmental conditions was devised by adapting the index proposed by Issler and Giugliani (19) for measuring poverty conditions in Brazil. Families were assigned to one of three groups based on the socioenvironmental level indicated by their total score: the first, second, and third tertiles corresponded to the score of 14-25, 26-31, and 32-39 respectively.

The second dimension included variables relating to the child's mother: mother's age (<20 years: adolescent; ≥20 years: adult), anaemia during pregnancy (yes or no), and educational level (basic integrated education, secondary education, or greater than secondary education). The last dimension comprised variables relating to the child: sex, age-group (<24 months; ≥24 months), diarrhoea during the previous three months (yes or no), duration of exclusive breastfeeding (≤3 months; >3 months), fully vaccinated relative to age (yes or no), and nutritional status as classified by the anthropometric indices.

The variables comprising each dimension of the hierarchical model were introduced into the model simultaneously, along with the variables that exhibited a significance level of ≤0.20 in the previous stage of the multivariate analysis. This significance level was chosen in order not to exclude potential confounders. Variables with p values of <0.05 in the last stage of the model were considered statistically associated with anaemia.

## RESULTS

Data from a total of 993 children aged 6-59 months, who resided on the nine islands of Cape Verde, were analyzed. There was a homogeneous distribution according to sex (49.7% male and 50.3% female); 31% were aged less than 24 months; 16.4% had experienced recent episodes of diarrhoea; and 57.5% had been exclusively breastfed for three months or less according to reports from their parents or caregivers. The overall prevalence of anaemia was 51.8% (95% CI 47.7-55.8). The most notable nutritional deficit was HAZ <−2 (13.3%); 10.2% of the children were overweight according to WHZ ≥2 (Table 1).

Among the children's mothers, 86.7% were 20 years old or over (adult); 54.6% had attended school up to end of basic integrated education; and 24.3% reported having had anaemia during the pregnancy (Table 2).

The distribution of the explanatory variables according to tertiles of household conditions is presented in Appendix

Bivariate analysis revealed that children below 24 months of age, children with recent episodes of diarrhoea (Table 1), and children whose mothers were below 20 years of age (Table 2) were significantly more likely to have anaemia than their counterparts.

In the first stage of the multivariate analysis (variables representing the socioeconomic and environmental conditions of families), we observed that anaemia was more likely prevalent in children of families classified into the first and the second tertiles of the household conditions than of those in the third tertile. Household conditions and maternal variables were introduced in the second stage; results showed that anaemia in children was more likely when their mothers were below the age of 20 years. In the third stage, child-related variables were considered, along with household conditions and maternal age. Household conditions, child's age-group (greater risk of anaemia in children below 24 months of age), and recent episodes of diarrhoea (p value borderline=0.06) remained associated with anaemia in the final model (Table 3). The interaction between these three variables was non-significant (interaction between age range and recent episodes of diarrhoea: p=0.82; between age range and household conditions: p=0.42; between recent episodes of diarrhoea and household conditions: p=0.79).

Table 4 summarizes the expected prevalence of anaemia for the variable categories that remained in the final model (the child's age-group, household conditions, and recent episodes of diarrhoea). Results indicate that the prevalence of anaemia could potentially reach 80% in children below 24 months, who had experienced recent episodes of diarrhoea and lived in the worst household conditions (first and second tertiles).

**Table 1.** Association of variables related to children with anaemia among children aged 6-59 months in Cape Verde (2009)

Variable	N	Frequency (%)	Prevalence of anaemia			p value
			%	OR	95% CI	
Sex						0.16
Male	513	49.7	54.3	1.22	(0.91-1.64)	
Female	480	50.3	49.2	1.0	(reference)	
Age-group						<0.001
<24 months	311	31.0	72.1	3.47	(2.35-5.14)	
≥24 months	682	69.0	42.6	1.0	(reference)	
Recent episodes of diarrhoea						0.03
Yes	163	16.4	61.2	1.57	(1.02-2.40)	
No	753	83.6	50.1	1.0	(reference)	
Exclusive breastfeeding						0.25
≤3 months	496	57.5	53.6	1.18	(0.88-1.5)	
>3 months	452	42.5	49.4	1.0	(reference)	
Complete vaccination						0.35
No	126	12.5	47.5	0.82	(0.54-1.24)	
Yes	867	87.5	52.4	1.0	(reference)	
WAZ						0.39
<2 z-score	35	3.2	54.3	1.13	(0.44-2.89)	
≥−2 and <2 z-score	910	91.8	51.1	1.0	(reference)	
≥2 z-score	48	5.0	62.1	1.56	(0.81-2.99)	
WHZ						0.37
<−2 z-score	56	5.2	47.9	0.75	(0.48-1.16)	
≥−2 and <2 z-score	828	84.5	51.3	1.0	(reference)	
≥2 z-score	95	10.3	56.1	1.21	(0.68-2.13)	
HAZ						0.12
<−2 z-score	122	13.3	60.9	1.53	(0.87-2.68)	
≥2 z-score	871	86.7	50.4	1.0	(reference)	

OR=Odds ratio; CI=Confidence interval; WAZ=Weight-for-age z-score; WHZ=Weight-for-height z-score; HAZ=Height-for-age z-score

## DISCUSSION

The results of the present study confirm the high prevalence of anaemia (51.8%) among children below the age of five years in Cape Verde. Thus, the incidence of anaemia appears to be constant, given the 2005 statistic of 52% prevalence among children in Cape Verde (3). Child's age (less than 24 months), recent episodes of diarrhoea, and the worse household condition were the variables that most contributed to the prevalence of anaemia. The combination of these factors could potentially result in anaemia in up to 80% of children.

Accordingly, anaemia is classified as a serious public-health issue in Cape Verde by WHO (5). However, the prevalence of anaemia observed in the present study was lower than the estimated mean for Africa in general, which is approximately 60% (1). In the same age-group, the prevalence was 82% in Benin and Mali (20) and 69.3% in Equatorial Guinea (21). It was also lower than that observed in Lake Albert, Uganda (68.9%) (22) but was higher than that found in Ivory Coast, also located in West Africa (39%) (23) and in other countries, such as East Timor and Mexico where prevalence rates are 31.3% (24) and 20.6% (25) respectively.

Cape Verde is considered to be one of the countries in the Economic Community of West African States (26), region with better socioeconomic conditions. In recent years, Cape Verde has been categorized as a middle-income country due to improvements in the country's economic conditions (27). This may explain the lower prevalence of anaemia compared to other countries in Africa that have poorer condition. Further, in most of these countries, cholera and malaria are recurrent diseases, rates of non-literacy are high, and armed conflicts displace the population and affect sanitation (26). The combination of these factors likely contributes to the high rate of anaemia among resident children.

**Table 2.** Associations of socioeconomic and environmental conditions and maternal variables with anaemia among children aged 6-59 months in Cape Verde (2009)

Socioeconomic and environmental conditions	N	Frequency (%)	Prevalence of anaemia			p value
			%	OR	95% CI	
Household conditions*						0.19
No information	365	43.2	49.8	1.17	(0.73-1.88)	
1st tertile	167	19.4	57.0	1.57	(0.88-2.76)	
2nd tertile	245	19.8	56.1	1.51	(0.91-2.54)	
3rd tertile	216	17.7	45.8	1.0	(reference)	
Educational level of head of household						0.52
Completed basic integrated education	621	63.4	51.1	0.90	(0.66-1.23)	
Secondary or more	362	36.6	53.6	1.0	(reference)	
Maternal characteristics						
Mother's educational level						0.48
Completed basic integrated education	554	54.6	50.7	0.90	(0.67-1.21)	
Secondary or more	434	45.5	53.3	1.0	(reference)	
Anaemia during pregnancy						0.9
Yes	222	24.3	52.4	1.02	(0.73-1.41)	
No	694	75.7	51.9	1.0	(reference)	
Mother's age						0.07
<20 years	124	13.3	63.8	1.73	(1.16-2.59)	
≥20 years	837	86.7	50.4	1.0	(reference)	

*No information included in the analysis because this variable presented a loss of information greater than 10%; CI=Confidence interval; OR=Odds ratio

Previous studies have demonstrated associations between anaemia and other indicators of poor socioeconomic conditions in Africa. Ngnie-Teta *et al.* (20) analyzed the risk of anaemia in relation to the family's wealth and observed a greater prevalence of anaemia among children who were at the lowest household living standard. Moreover, Custodio *et al.* (21) found that children who lived in households with lower socioeducational levels were at greater risk of anaemia. Studies conducted in other countries have also shown associations between anaemia and unfavourable living and sanitary conditions (28).

The indicated increased risk of anaemia in children below the age of 24 months is consistent with findings from other countries (29). This vulnerability is likely due to the increased need for iron at this age and inadequate introduction of iron-rich foods after weaning. In Cape Verde, particularly the foods most commonly consumed by the population are cereals (30) which are not adequate sources of iron.

This is the first study showing the magnitude of anaemia in children below 24 months of age in Cape Verde; a very high prevalence (72.1%) has been indicated. It is worth noting, however, that no specific action has yet been implemented in Cape Verde for combating anaemia in the most vulnerable age-group since the prophylactic distribution of ferrous sulphate is limited to school-age children.

Another variable significantly associated with childhood anaemia was recent episodes of diarrhoea, congruent with the findings of other studies in developing countries (31,32). Agho *et al.* (24) observed lower concentrations of haemoglobin among children of East Timor, who had reportedly experienced recent episodes of diarrhoea. Diarrhoea is associated with iron loss via the faeces and decreased absorption of the nutrients necessary for maintaining normal haemoglobin levels in the blood (32). Episodes of diarrhoea may indicate the presence of infectious parasites, which may occur more frequently in unhealthy environments, such as those exposed to the most unfavourable socioenvironmental conditions. One study conducted in Cape Verde demonstrated a high incidence of intestinal parasitic infections in all age-groups, reaching 59.17% in children aged 0-5 year(s) (33). This was attributed to poor sanitary and environmental conditions, which may influence the nutritional status of children (34).

**Table 3.** Results of logistic regression of the hierarchical multivariate analysis for children aged 6-59 months in Cape Verde (2009)

Variable	1st stage		2nd stage		3rd stage	
	OR 95% CI	p value	OR 95% CI	p value	OR 95% CI	p value
Household conditions		0.19		0.06		0.05
No information	1.18 (0.74-1.90)		1.33 (0.79-2.25)		1.14 (0.69-1.88)	
1st tertile	1.58 (0.90-2.73)		1.86 (1.06-3.32)		1.94 (1.01-3.76)	
2nd tertile	1.51 (0.87-2.56)		1.83 (1.04-3.21)		1.64 (0.90-2.97)	
3rd tertile	1.0 (reference)		1.0 (reference)		1.0 (reference)	
Educational level of head of household		0.22				
Completed basic integrated education	0.88 (0.64-1.21)		-		-	
Secondary or more	1.0 (reference)		-		-	
Mother's educational level				0.93		
Completed basic integrated education	-		0.98 (0.70-1.37)			
Secondary or more	-		1.0 (reference)			
Anaemia during pregnancy				0.84		
Yes	-		0.96 (0.67-1.37)		-	
No	-		1.0 (reference)		-	
Mother's age				0.04		0.35
<20 years	-		1.58 (1.01-2.49)		1.25 (0.77-2.02)	
≥20 years	-		1.0 (reference)		1.0 (reference)	
Sex						0.19
Male	-		-		1.23 (0.89-1.71)	
Female	-		-		1.0 (reference)	
Age-group						<0.001
<24 months	-		-		3.29 (2.09-5.18)	
≥24 months	-		-		1.0 (reference)	
Recent episodes of diarrhoea						0.06
Yes	-		-		1.53 (0.97-2.40)	
No	-		-		1.0 (reference)	
Exclusive breastfeeding						0.47
≤3 months	-		-		1.14 (0.79-1.65)	
>3 months	-		-		1.0 (reference)	
Complete vaccination						0.15
No	-		-		0.72 (0.46-1. 13)	
Yes	-		-		1.0 (reference)	
WAZ						0.74
<−2 z-scores	-		-		1.22 (0.38-3.95)	
≥−2 and <2 z-scores	-		-		1.0 (reference)	
≥2 z-scores	-		-		1.58 (0.75-3.33)	
WHZ						0.81
<−2 z-scores	-		-		0.79 (0.38-1.66)	
≥−2 and <2 z-scores	-		-		1 (reference)	
≥2 z-scores	-		-		0.90 (0.47-1.74)	
HAZ						0.09
<−2 z-scores	-		-		1.58 (0.92-2.71)	
≥2 z-scores	-		-		1.0 (reference)	

OR=Odds ratio; CI=Confidence interval; WAZ=Weight-for-age z-score; WHZ=Weight-for-height z-score; HAZ=Height-for-age z-score

Previous studies have implicated malaria as an important risk factor of anaemia, especially in sub-Saharan African countries (8,31). It was not possible in the present study to evaluate the role of malaria in anaemia as the incidence of malaria infection is low in Cape Verde (rate of 0.9 per 10,000) (11).

As an important nutritional deficiency, anaemia may be associated with anthropometric indicators of nutritional deficit. However, in the present study, weight-height deficits were not associated with the prevalence of anaemia, in contrast to previous reports on other African countries (20,31). The absence of this association found in the present study may have resulted from the difference in the prevalence of height-for-age deficits observed in previous studies (25%) and the present study (13.3%).

### Limitations

This study has some limitations. First, the cross-sectional design did not allow any causal relationships to be established. Second, children who were excluded from the analysis due to age were not characterized as it was not possible to access birth registration documents of these children. Furthermore, the large number of families that compose ‘no information’ group in the household conditions variable (near 50%) can bring some bias in interpreting the results about the association between anaemia and household conditions.

### Conclusions

The present study demonstrates that the prevalence of anaemia in children below the age of 5 years in Cape Verde remains high and especially affects children below 24 months of age living in unfavourable household and socioenvironmental conditions and presenting recent episodes of diarrhoea.

**Table 4.** Expected prevalence of anaemia in children in Cape Verde, aged 6-59 months, according to the category of the variables that remained associated with anaemia in the 3rd stage of the hierarchical multivariate analysis (2009)

Variable	Household conditions			
	1st tertile	2nd tertile	3rd tertile	No information
<24 months				
With diarrhoea	81.5	80.8	74.3	74.1
Without diarrhoea	77.7	76.8	69.4	69.2
≥24 months				
With diarrhoea	54.0	52.8	43.4	43.2
Without diarrhoea	48.0	46.8	37.6	37.4

Although a national programme to fortify flour with iron and folic acid has been in operation since 2008, this strategy may not have a direct impact on children of the most vulnerable age-group. As recommended by the World Health Organization (5), the preferred anaemia-prevention strategy for this age-group is ferrous sulphate supplementation. The promotion of exclusive breastfeeding until the age of 6 months, diversification of the food-complementation diet, fortification of foods commonly consumed by Cape Verdean children, improvement of living conditions, and health education programmes should also be other strategies to improving the health of children below 5 years of age in Cape Verde

This study may support the implementation of programmes or actions to prevent anaemia and improve the general health of children in Cape Verde.

## ACKNOWLEDGEMENTS

We thank the Government of Cape Verde, especially the Ministry of Environment, Rural Development and Marine Resources, for financing this project. We further acknowledge the Federal University of Rio de Janeiro for the technical and scientific support given to the Survey on the Prevalence of Anemia and Associated Factors among children below the age of 10 years, which generated data for this study, and the CNPq (National Council for Scientific and Technological Development) for the scholarship award.

## Appendix

**Appendix.** Frequency of demographics and other explanatory variables according to tertiles of household conditions

Variable	1st tertile	2nd tertile	3rd tertile	No information
Sex				
Male	18.1	20.6	17.3	44.1
Female	20.6	19.0	18.1	42.3
Age-group				
<24 months	18.7	17.7	18.0	45.7
≥24 months	19.7	20.8	17.5	42.1
Mother's age				
<20 years	16.3	32.4	11.9	39.4
≥20 years	19.4	17.5	18.5	44.5
Anaemia during pregnancy				
Yes	19.5	21.4	16.6	42.5
No	19.3	18.2	17.7	44.8
Recent episodes of diarrhoea				
Yes	18.1	18.9	19.9	43.0
No	20.4	20.2	16.6	42.8
Exclusive breastfeeding				
≤3 months	23.3	17.3	15.2	44.1
>3 months	15.6	20.6	20.3	43.5
Complete vaccination				
Yes	18.1	20.6	17.8	43.5
No	28.3	13.9	17.0	40.7
HAZ				
<−2 z-score	29.0	18.6	14.7	37.7
≥−2 z-score	17.9	20.0	18.1	44.0
WAZ				
<−2 z-score	20.3	15.6	3.3	60.8
≥−2 and <2 z-score	19.2	19.7	18.1	42.9
≥2 z-score	21.0	23.5	19.0	36.5
WHZ				
<−2 z-score	18.2	26.5	17.7	37.5
≥−2 and <2 z-score	19.5	18.6	18.0	44.0
≥2 z-score	13.3	28.4	16.0	42.4

HAZ=Height-for-age z-score; WAZ=Weight-for-age z-score; WH=Weight-for-height z-score

## References

[1] World Health Organization (2008). Worldwide prevalence of anaemia 1993-2005: WHO global database on anaemia.

[2] Magalhães RJS, Clements ACA (2011). Spatial heterogeneity of haemoglobin concentration in preschool-age children in sub-Saharan Africa. Bull Word Health Organ.

[3] Cape Verde Ministério da Saúde. Inquérito Demográfico e de Saúde Reprodutiva (IDSR) 2005 2008 Praia Instituto Nacional de Estatística, Ministério da Saúde, República De Cabo Verde 312 (http://www.ine.cv/actualise/publicacao/files/f837f3ed-39c8-46d2-a112-9e59196f4fe0CV_IDSR-II.pdf, accessed on 23 March 2008).

[4] Cape Verde Ministério da Saúde. Fundo das nações unidas para a infância: UNICEF. Saúde das crianças menores de cinco anos em Cabo 1996 Praia Ministério da Saúde, Government of Cape Verde 131 p. (http://pt.scribd.com/doc/134117704/Livro-Saude-das-Criancas-Menores-de-5-anos-em-Cabo-Verde-Minist-Saude-e-UNICEF-1996, accessed on 20 March 2008). [Portuguese]

[5] World Health Organization (2001). Iron deficiency anaemia: assessment, prevention and control; a guide for programme managers.

[6] Sachdev H, Gera T, Nestel P (2005). Effect of iron supplementation on mental and motor development in children: systematic review of randomised controlled trials. Public Health Nutr.

[7] World Health Organization (2001). Iron deficiency anaemia: assessment, prevention, and control. A guide for programme managers..

[8] Korenromp EL, Armstrong-Schellenberg JRM, Williams BG, Nahlen BL, Snow RW (2004). Impact of malaria control on childhood anaemia in Africa—a quantitative review. Trop Med Int Health.

[9] Instituto Nacional de Estatística [Census 2010]. IV Recenseamento geral da população e habitação: resumo dos principais resultados por meio de residência e concelho 2011 Praia Instituto Nacional de Estatística, República de Cabo Verde 40 (http://www.ine.cv/censo/censo2010.aspx, accessed on 15 October 2013). [Portuguese]

[10] Instituto Nacional de Estatística Inquérito dos Indicadores do Bem Estar (QUIBB). [Survey of indicators of welfare] 2007 Praia Instituto Nacional de Estatística, República de Cabo Verde 155 (http://www.ine.cv/publicacoes/details.aspx?d=249, accessed on 10 April 2010). [Portuguese]

[11] Cape Verde Ministério da Saúde. [Statistical Report] 2011 Praia Republica de Cabo Verde 118 (www.minsaude.gov.cv/index.php/…/doc…/240-relatorio-estatistico-2011, accessed on 15 June 2013). [Portuguese]

[12] Lwanga SK, Lemeshow S (1991). Sample size determination in health studies: a practical manual.

[13] Be WKM, Kerkkamp HEM, Booij LHDJ (1991). Hemocue—a new haemoglobinometer in the clinic. Eur J Anaesthesiol.

[14] Habicht JP (1974). [Standardization of quantitative epidemiological methods in the field]. Bol Oficina Sanit Panam.

[15] World Health Organization (2006). WHO child growth standards: length/height-for-age, weight-for-age, weight-for-length, weight-for-height and body mass index-for-age; methods and development.

[16] Pearce N (2004). Effect measures in prevalence studies. Environ Health Perspect.

[17] de Silva LSM, Giuglian ERJ, de Castro Aerts DRG (2001). [Prevalence and risk factors for anemia among children in Brazil]. Rev Saúde Pública.

[18] Shyamsundar P (2002). Poverty: environment indicators.

[19] Issler RM, Giugliani ER (1997). [Identification of the groups most vulnerable to infant malnutrition through the measuring of poverty level]. J Pediatr (Rio J).

[20] Ngnie-Teta I, Receveur O, Kuate-Defo B (2007). Risk factors for moderate to severe anemia among children in Benin and Mali: insights from a multilevel analysis. Food Nutr Bull.

[21] Custodio E, Descalzo MA, Roche J, Sánchez I, Molina L, Lwanga M (2008). Nutritional status and its correlates in Equatorial Guinean preschool children: results from a nationally representative survey. Food Nutr Bull.

[22] Green HK, Sousa-Figueiredo JC, Basáñez M-G, Betson M, Kabatereine NB, Fenwick A (2011). Anaemia in Ugandan preschool-aged children: the relative contribution of intestinal parasites and malaria. Parasitology.

[23] Asobayire FS, Adou P, Davidsson L, Cook JD, Hurrell RF (2001). Prevalence of iron deficiency with and without concurrent anemia in population groups with high prevalence of malaria and other infections: a study in Côte d’Ivoire. Am J Clin Nutr.

[24] Agho KE, Dibley MJ, D’Este C, Gibberd R (2008). Factors associated with haemoglobin concentration among Timor-Leste children aged 6-59 months. J Health Popul Nutr.

[25] de la Cruz-Góngora V, Villalpando S, Rebollar R, Shamah-Levy T, Méndez-Gómez Humarán I (2012). Nutritional causes of anemia in Mexican children under 5 years. Results from the 2006 National Health and Nutrition Survey. Salud Publica Mex.

[26] West African Health Organization Strategic plan 2009-2013 2008 Bobo-Dioulasso West African Health Organization 52 (http://www.wahooas.org/IMG/pdf/PS-2009-2013-EN_q_web.pdf, accessed on 20 June 2010).

[27] Cape Verde One programme in Cape Verde 2008/2011 English version 2009. Annex 2. 2009 Praia Republic of Cape Verde 23 (http://www.undg.org/docs/10241/2-ONE-PROGRAMME-in-Cape-Verde---English-version.June09.pdf, accessed on 22 October 2013).

[28] Leal LP, Batista Filho M, de Lira PI, Figueiroa JN, Osório MM (2012). Temporal trends and anaemia-associated factors in 6- to 59-month-old children in Northeast Brazil. Public Health Nutr.

[29] Austin AM, Fawzi W, Hill AG (2012). Anaemia among Egyptian Children between 2000 and 2005: trends and predictors. Matern Child Nutr.

[30] Cabo Verde Instituto Nacional de Estatística. Avaliação da situação de insegurança alimentar em Cabo Verde: análise dos dados de consumo alimentar 2007 Praia Instituto Nacional de Estatística, Ministério do Ambiente e Agricultura, República de Cabo Verde 45 (http://www.ine.cv/actualise/publicacao/files/e600a95e-c0c9-45fd-b721-223ccb2031edRELAT%C3%93RIOFINALANALISE NUTRI%C3%87%C3%83O.pdf, accessed on 20 April 2010). [Portuguese]

[31] Desai MR, Terlouw DJ, Kwena AM, Phillips-Howard PA, Kariuki SK, Wannemuehler KA (2005). Factors associated with hemoglobin concentrations in pre-school children in Western Kenya: cross-sectional studies. Am J Trop Med Hyg.

[32] Hassan K, Sullivan KM, Yip R, Woodruff BA (1997). Factors associated with anemia in refugee children. J Nutr.

[33] Cabo Verde Ministere de la Sante du Cap Vert. Organisation Mondiale de la Sante. [Survey on the prevalence of intestinal parasites in schools and gardens in Cape Verde (November 2004-April 2005). Papport mission] 2005 Praia República de Cabo Verde 31 (http://www.afro.who.int/pt/cabo-verde/cabo-verde-publicacoes.html, accessed on 20 April 2010). [French]

[34] Osazuwa F, Ayo OM, Imade P (2011). A significant association between intestinal helminth infection and anaemia burden in children in rural communities of Edo state, Nigeria. N Am J Med Sci.

